# Characterisation of metabolites of the putative cancer chemopreventive agent quercetin and their effect on cyclo-oxygenase activity

**DOI:** 10.1038/sj.bjc.6602091

**Published:** 2004-08-03

**Authors:** D J L Jones, J H Lamb, R D Verschoyle, L M Howells, M Butterworth, C K Lim, D Ferry, P B Farmer, A J Gescher

**Affiliations:** 1 Cancer Biomarkers and Prevention Group, Department of Biochemistry, Biocentre, University of Leicester, Leicester LE1 7RH, UK; 2 Cancer Biomarkers and Prevention Group, Department of Cancer Studies, University of Leicester, Leicester LE1 7RH, UK; 3 Medical Research Council Toxicology Unit, University of Leicester, Leicester LE1 9HN, UK; 4 Medical Research Council Bioanalytical Science Group, School of Biological and Chemical Sciences, Birkbeck College, University of London, Malet Street, London WC1E 7HX, UK; 5 The Royal Wolverhampton Hospitals NHS Trust, New Cross Hospital, Wednesfield Road, Wolverhampton WV10 0QP, UK

**Keywords:** cancer chemoprevention, cyclo-oxygenase, metabolism, quercetin

## Abstract

Quercetin (3,5,7,3′,4′-pentahydroxyflavone) is a flavone with putative ability to prevent cancer and cardiovascular diseases. Its metabolism was evaluated in rats and human. Rats received quercetin via the intravenous (i.v.) route and metabolites were isolated from the plasma, urine and bile. Analysis was by high-performance liquid chromatography and confirmation of species identity was achieved by mass spectrometry. Quercetin and isorhamnetin, the 3′-*O*-methyl analogue, were found in both the plasma and urine. In addition, several polar peaks were characterised as sulphated and glucuronidated conjugates of quercetin and isorhamnetin. Extension of the metabolism studies to a cancer patient who had received quercetin as an i.v. bolus showed that (Quercetin removed) isorhamnetin and quercetin 3′-*O*-sulphate were major plasma metabolites. As a catechol, quercetin can potentially be converted to a quinone and subsequently conjugated with glutathione (GSH). Oxidation of quercetin with mushroom tyrosinase in the presence of GSH furnished GSH conjugates of quercetin, two mono- and one *bis*-substituted conjugates. However, these species were not found in biomatrices in rats treated with quercetin. As cyclo-oxygenase-2 (COX-2) expression is mechanistically linked to carcinogenesis, we examined whether quercetin and its metabolites can inhibit COX-2 in a human colorectal cancer cell line (HCA-7). Isorhamnetin and its 4′-isomer tamarixetin were potent inhibitors, reflected in a 90% decrease in prostaglandin E-2 (PGE-2) levels, a marker of COX-2 activity. Quercetin was less effective, with a 50% decline. Quercetin 3- and 7-*O*-sulphate had no effect on PGE-2. The results indicate that quercetin may exert its pharmacological effects, at least in part, via its metabolites.

Naturally occurring flavonoids in the diet are associated with several beneficial health effects and understanding the mechanisms underlying these effects has become the focus of much research. Quercetin (3,5,7,3′,4′-pentahydroxyflavone, for structure see Figure 1

**Figure 1:**
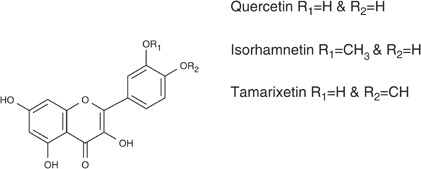
Structures of quercetin and its metabolites.

) is a prime example of such a flavonoid. Its glycosylated form occurs in kale, French beans, broccoli, apples and especially in onions, with an abundance as high as a quarter to half a gram per kg (Hertog *et al*, 1992). On ingestion with the diet, quercetin glycosides are rapidly hydrolysed to generate quercetin.

Epidemiological evidence links diets rich in quercetin with decreased incidence of cardiovascular and neoplastic diseases (Hertog *et al*, 1993, 1994, 1995; Hertog and Hollman, 1996; Keli *et al*, 1996; Le Marchand *et al*, 2000). From the mechanistic standpoint, quercetin has been shown to interact with cellular processes in numerous ways (for a review see Nijveldt *et al*, 2001). Recently, cyclo-oxygenase (COX) enzymes, especially COX-2, have been causally implicated in the early changes associated with carcinogenesis in a number of tissues, among which the colorectal tract has been studied most extensively (Marnett and Dubois, 2002). A noteworthy mechanistic facet of quercetin is its ability to interfere with COX by inhibiting COX-2 promoter activity (Mutoh *et al*, 2000), COX-2 protein expression (Raso *et al*, 2001) and COX enzyme activity (Formica and Regelson, 1995). In the light of its interesting biological properties germane to anticarcinogenesis, quercetin has been subjected to a phase I clinical trial in cancer patients, with the aim to develop it as a cancer chemopreventive or antineoplastic agent (Ferry *et al*, 1996). With respect to potential detrimental effects on health by quercetin, it has been suggested to possess mutagenic and carcinogenic properties (MacGregor and Jurd, 1978; Dunnick and Hailey, 1992), and at high doses there were indications of toxicity in humans (Ferry *et al*, 1996).

Quercetin shares with many naturally occurring polyhydroxylated molecules poor bioavailability (Hollman and Katan, 1997), which is probably a consequence of its rapid metabolic transformation in the liver and gastrointestinal tract. Evidence is accumulating that metabolites of dietary polyphenols may mediate, or substantially contribute to, the pharmacological efficacy of the parent molecule, and thus explain efficacy despite the apparent low bioavailability, as is observed with 1,3,4′-trihydroxy-*trans*-stilbene (resveratrol), which is found in grapes (Gescher and Steward, 2003). Therefore, identifying the metabolites of dietary components such as quercetin and resveratrol and defining their contribution to the pharmacological and biological effects of the parent molecule is of great importance. The fate in the mammalian organism of the dietary progenitor glycosides of quercetin, such as rutin and quercetrin, has been more extensively studied than the metabolism of quercetin itself, when administered in aglyconic form. Quercetin is oxidatively degraded to nonflavone phenols, probably by intestinal bacteria (Booth *et al*, 1956; Douglass and Hogan, 1958; Petrakis *et al*, 1959). Moreover, these fission products have been shown to possess biological activity (Merfort *et al*, 1996). Most importantly, quercetin has been shown to undergo conjugation with glucuronide and sulphate at one or more of the five hydroxyl moieties of the flavone molecule and methylation at positions 3′or 4′, thus generating isorhamnetin (3′-*O*-methylquercetin) and tamarixetin (4′-O-methylquercetin). These conjugates have been characterised in rat urine, bile and plasma (Ueno *et al*, 1983; Manach *et al*, 1997; Piskula and Terao, 1998). A quercetin sulphate and two quercetin sulphate-glucuronides were also found in the perfused rat liver (Shali *et al*, 1991). Metabolites were also identified in human plasma following the ingestion of a complex meal rich in plant products (Manach *et al*, 1998, Day *et al*, 2001). Two pharmacokinetic studies of authentic quercetin in humans established that it is rapidly cleared from the organism (Gugler *et al*, 1975; Ferry *et al*, 1996).

In the light of the interest in quercetin as a potential cancer chemopreventive or chemotherapeutic agent, we reinvestigated its metabolism in the rat using mass spectrometric methods of chemical identification. A particular aim of the study was to compare the qualitative pattern observed in rat plasma with that found in a human who had undergone treatment with quercetin in a clinical trial (Ferry *et al*, 1996). In the light of the indications of its potential nephrotoxicity demonstrated in that trial, we tested the potential of quercetin to undergo biotransformation via conjugation with glutathione (GSH) to a proximate nephrotoxicant, analogous to the nephrotoxicity associated with hydroquinone (Peters *et al*, 1997), bromohydroquinone (Monks *et al*, 1985), 17*β*-estradiol (Butterworth *et al*, 1997) or haloalkenes (Iverson *et al*, 1996). Previous reports have indicated that GSH conjugates are formed *in vitro* (Awad *et al*, 2000, 2001; Boersma *et al*, 2000; Galati *et al*, 2001). Furthermore, mindful of the fact that COX activity is a potential mechanistic target of quercetin, we compared its effect on cellular prostaglandin E-2 (PGE-2) production with that of representative quercetin metabolites. Overall the work was designed to contribute to the database required for the optimisation of the clinical development of quercetin as a potential cancer chemopreventive and/or chemotherapeutic agent.

## MATERIALS AND METHODS

### Materials

The following materials were purchased from the indicated sources. Quercetin, rutin mushroom tyrosinase, high-performance liquid chromatography (HPLC) grade dimethyl sulphoxide (DMSO), ammonium acetate: Sigma (Poole, UK); HPLC grade methanol: Fisher (Loughborough, UK); isorhamnetin, tamarixetin, quercetin 3-*O*-sulphate: Extrasynthese (Genay, France); and glycerol formal: Fluka (Poole, UK). Human colon adenoma cells (HCA-7, passage number 29) were obtained from Dr S Kirkland (Imperial College, London, UK).

### Animals, treatments and incubation conditions

Male F344 rats (200–250 g) were used. For metabolite analysis in the plasma, animals were anaesthetised with pentobarbital (6 mg per rat, i.p.), and quercetin (6.25 mg kg^−1^) dissolved in glycerol formal and water 1 : 1 was injected intravenous (i.v.) via the lateral tail vein (injection volume: 100 *μ*l). After 5 min blood was collected by cardiac puncture. For the analysis of metabolites in bile, the quercetin dose was 12.5 mg kg^−1^ (i.v.). For the study of urinary metabolites, quercetin dissolved in DMSO (0.5 ml kg^−1^) was given by gavage (2.5 g kg^−1^), rats were transferred to metabolism cages and urine was collected for up to 24 h postadministration. For the analysis of metabolites in the bile, rats were anaesthetised (pentobarbitol) and the bile duct was cannulated. Ambient body temperature was maintained using a heat lamp. Control animals received the vehicle only. Experiments were conducted as stipulated by Project Licence 80/1250 granted by the UK Home Office. The experimental design was vetted and approved by the Leicester University Ethical Committee for Animal Experimentation and meet the standards required by the UKCCCR guidelines (Workman *et al*, 1998).

In order to generate reactive oxidation products of quercetin *in vitro*, quercetin and GSH (1 mM each) were incubated for 1 h (37°C) with mushroom tyrosinase in ammonium acetate buffer (0.1 M, pH 7.4).

### Quercetin metabolism in a human

Blood samples were obtained from a cancer patient, at the Queen Elizabeth Hospital (Birmingham, UK), who no longer responded to standard therapy and had been recruited into a phase I clinical study of quercetin. This trial has been described in detail before (Ferry *et al*, 1996). Quercetin formulated in DMSO was infused i.v. (250 mg m^−2^ for 5 min) and blood was obtained up to 2 h postadministration. The plasma was separated and stored at −80°C.

### Sample preparation and HPLC analysis

Aliquots of plasma, urine, bile or tyrosinase incubate were mixed with twice the volume of DMSO/methanol (1 : 4, v v^−1^). Kaempferol (internal standard) was added and the mixture was vortexed and centrifuged (17 060 **g**, 15 min). The supernatant was removed, diluted with water (1 : 1) and injected onto the HPLC column (injection volume 50 *μ*l). Hydrolysis of glucuronide and sulphate conjugates of quercetin in the plasma or bile was performed as described by Ader *et al* (2000). In short, *β*-glucuronidase solution (500 U in 0.1 M ammonium acetate pH 6.8) also containing sulphatase was added to an aliquot (0.1 ml) of biofluid (acidified with 20 *μ*l of 0.5 M acetic acid). Incubations were carried out for 30 min at 37°C, which furnished maximal deconjugation. Following incubation samples were extracted for analysis.

High-performance liquid chromatography analysis was performed on a Varian Prostar system, which comprised of a UV detector (model 310), solvent delivery system (model 230) and an autosampler (model 410) with a 100 *μ*l loop. Detection was by UV at 375 nm. Analysis of the human plasma samples was performed as described previously (Jones *et al*, 1998). Analysis of extracts of rat plasma, urine or bile involved a gradient mobile phase system (75% 0.1 M ammonium acetate in methanol, decreased linearly to 55% for 10 min and to 45% for a further 20 min, at which it remained for a further 5 min). The flow rate was 1 ml min^−1^. Separation was achieved on a Hypersil BDS C_18_ column (250 × 4.6 mm^2^ i.d., 5 *μ*m particle size; Hypersil, Runcorn, UK). The mobile phase used for the analysis of GSH conjugates consisted of the same start eluent, but the content of ammonium acetate decreased to 55% for 20 min, at which it remained for a further 50 min.

For the quantitation of quercetin in plasma stock solutions (10 mg ml^−1^) of quercetin and kaempferol (internal standard) were made up (DMSO), and the calibration curve was constructed with five concentrations of quercetin (final concentrations 1, 2, 20, 100 and 200 *μ*g ml^−1^). The recovery of quercetin from the plasma was 90%, and the concentrations described in the results were corrected correspondingly.

### Mass spectrometry (MS)

A Quattro Bio-Q triple stage mass spectrometer (Micromass, Manchester, UK) fitted with pneumatically assisted electrospray (pnESI) was coupled to a Varian 9012 pump (flow rate: 1 ml min^−1^). A tee-piece was incorporated postcolumn to give a 1 : 7 split, so that approximately 140 *μ*l min^−1^ entered the source region of the mass spectrometer. Standards were infused into the mass spectrometer using a Harvard syringe pump (10 *μ*l min^−1^). Typically, a capillary voltage of 3.78 kV, HV lens voltage of 0.2 kV and a cone voltage of 46 V was used. Source temperature was 110°C. Two types of experiments were conducted: (i) a scan of 50–1300 *m/z*, from which ion chromatograms were extracted postrun and (ii) LC-MS/MS in which specific ions underwent dissociation in the collision cell. Collision gas was argon. All mass spectra were acquired in the negative ion mode.

### PGE-2 levels in HCA-7 cells

HCA-7 cells were routinely cultured in Dulbecco's MEM medium with Glutamax (Gibco, Invitrogen Corp., UK) supplemented with 10% foetal calf serum and penicillin/streptomycin (1000 and 500 U ml^−1^, respectively). Cells (10^5^ well^−1^) were incubated for 6 h with quercetin (1–75 *μ*M) or quercetin metabolites (10 *μ*M) dissolved in DMSO. Control cultures contained DMSO only. An aliquot (1 ml) of cellular supernatant was removed from each of the plates after 6 h and analysed in duplicate for PGE-2 content using a kit, which employs an ELISA immunoassay (Cayman Chemical Comp., MI, USA). Experiments were repeated three times. An initial experiment involved the incubation of 50 *μ*M quercetin and taking samples at both 6 and 24 h.

## RESULTS

### Metabolites of quercetin in the rat

**Figure 2:**
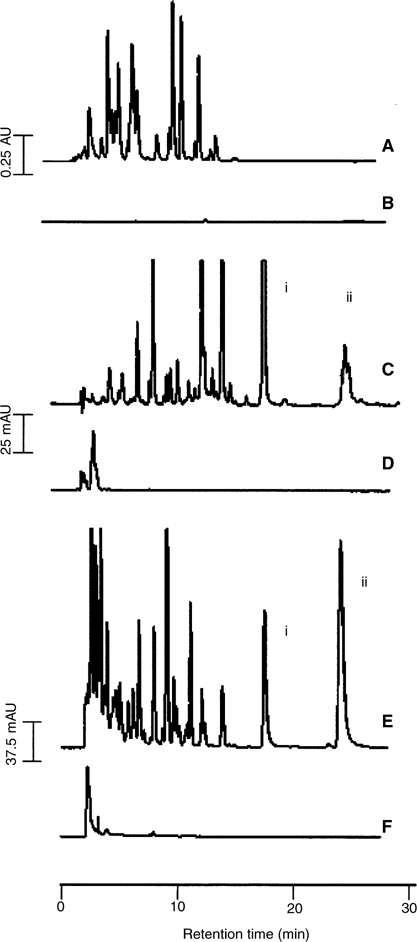
High-performance liquid chromatography chromatograms of extracts of bile (**A** and **B**), plasma (**C** and **D**) or urine (**E** and **F**) from control rats (**B**, **D** and **F**) or rats that had received quercetin either 12.5 (**A**) or 6.25 mg kg^−1^ (**C**) and 2.5 g kg^−1^ (**E**) via the i.v. (**A** and **C**) or oral (**E**) routes. Bile was collected prior to (control) and for 20 min after the administration of quercetin; plasma samples were collected 5 min after administration of quercetin; urine samples were pooled over 8 h. Control (untreated) animals received the vehicle only via the appropriate administration route. Symbols i and ii denote retention times of quercetin and isorhamnetin, respectively. AU=absorbance units. For details of sample preparation and chromatographic analysis see Materials and Methods. The chromatograms shown are representative of extracts obtained from three separate animals.

High-performance liquid chromatography analysis of plasma and urine samples from rats, which had received quercetin (6.25 mg kg^−1^) via the i.v. route, contained quercetin (retention time 17.5 min, peak i in Figure 2

) and a major metabolite (retention time 26 min, peak ii in Figure 2), which coeluted with isorhamnetin. Peak identification was confirmed by both on-line LC-MS and LC-MS of collected peak fractions, furnishing deprotonated molecule ions of *m/z* 301 for quercetin and *m/z* 315 for isorhamnetin.

Extracts of plasma, urine or bile from quercetin-treated animals characteristically gave 15 to 18 extra peaks (between the retention times of 5 and 16 min) that were absent from extracts of biomatrices obtained from untreated animals (Figure 2). On the basis of their polarity, these species were hypothesised to constitute sulphate and glucuronide conjugates of quercetin, which was confirmed by their disappearance on treatment with sulphatase and *β*-glucuronidase and a concomitant increase in the size of the quercetin and isorhamnetin peaks (result not shown). To confirm this conclusion, the extracts were analysed by LC-MS and ion chromatograms extracted postrun. Based on the pseudomolecular ion, 10 different types of conjugate species were detected (Table 1

**Table 1:** Deprotonated molecular ions [M–H]^−^ and HPLC retention times of metabolites of quercetin identified by selected ion monitoring in the plasma, urine and bile of rats that received quercetin (6.25 mg kg^−1^ i.v.)

**Metabolite**	***m/z***	**Number of isomers**	**Retention time (min)**
Quercetin glucuronides	477	5	7.32, 8.76, 9.85, 10.77, 13.08
Quercetin sulphates	381	2	8.67, 14.92
Quercetin sulphate-glucuronides	557	5	4.90, 5.76, 6.40, 6.71, 6.97
Quercetin *bis*-sulphates	461	2	11.52, 12.30
Quercetin *bis*-glucuronides	653	2	5.01, 5.45
Isorhamnetin glucuronides	491	4	10.08, 10.72, 12.16, 13.48
Isorhamnetin sulphates	395	2	9.54, 14.98
Isorhamnetin *bis*-glucuronides	667	2	4.61, 5.30
Isorhamnetin sulphate-glucuronide	571	5	5.22, 5.86, 6.51, 6.80, 7.23
Isorhamnetin *bis*-sulphate	475	4	9.54, 11.18, 11.29, 11.61

**Figure 3:**
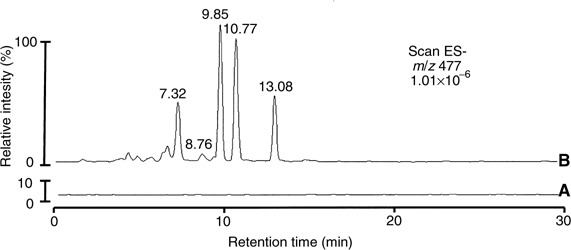
Extracted ion chromatogram (*m/z* 477) of (**A**) extract of bile from control rat and (**B**) quercetin glucuronides from bile of rats, which received quercetin (12.5 mg kg^−1^ i.v.). Retention times are those noted in Table 1.

HPLC=high-performance liquid chromatography.

). In order to illustrate the findings in Table 1, Figure 3

shows an extracted ion chromatogram of *m/z* 477 of quercetin glucuronides found in the bile. Allocation of these structures to chromatographic peaks furnished the following number of positional isomers: two isomers each of quercetin sulphate, quercetin *bis*-sulphate, quercetin *bis*-glucuronide, isorhamnetin sulphate and isorhamnetin *bis*-glucuronide, four isomers of isorhamnetin *bis*-sulphate, four isomers of isorhamnetin glucuronide, and five isomers each of quercetin sulphate-glucuronide and isorhamnetin sulphate-glucuronide. Of the two isomeric quercetin sulphates found, the species characterised by a retention time of 8.7 min coeluted with authentic quercetin 3′-*O*-sulphate. Characterisation of urine extracts by LC-MS/MS led to the identification of six species, quercetin, isorhamnetin, quercetin sulphate, quercetin glucuronide, isorhamnetin glucuronide and quercetin *bis*-glucuronide (Table 2

**Table 2:** Mass spectral properties of quercetin and five quercetin metabolites in the urine

**Metabolite**	**Retention time (min)^a^**	***m/z* (relative intensity)**
Quercetin	19.2	301(50), 151 (100), 273 (15), 179 (16), 135 (30), 121 (29), 107 (50)
Isorhamnetin	26.7	315 (10), 300 (90), 271(20), 151 (100), 107 (90)
Quercetin sulphate	15.6	381 (60), 301 (100), 179 (20), 151 (50), 107(30)
Quercetin glucuronide	9.3	477(15), 301 (100), 179 (12), 151(20)
Isorhamnetin glucuronide	12.6	491(10), 315(50), 300(100), 271(10), 151(10)
Quercetin *bis*-glucuronide	3.8	653 (100), 477 (20), 301 (10), 151 (10)

a Owing to the fact that this analysis was conducted over a considerable time after that shown in Table 1, the retention times shown for the sulphates and glucuronides differ from those described in Table 1 by up to 0.8 min.

). Cochromatography suggested that the urinary quercetin sulphate was quercetin 3′-*O*-sulphate.

In an attempt to obtain preliminary information on quercetin levels, quercetin was quantitated in the plasma. Shortly after administration (5 min) quercetin levels in the plasma were 13.7±4.1 *μ*M (*n*=6), beyond which time the agent disappeared rapidly. Quercetin conjugates were present until the 90 min time point postadministration. When plasma samples were subjected to enzymatic hydrolysis, the level of total quercetin, that is, conjugated and unconjugated, at the 5 min time point increased to 76.9±11.4 *μ*M. These results suggest that at this early time point species derived from quercetin circulating in the blood were predominantly quercetin glucuronides and sulphates. Bile obtained from these rats showed the presence of quercetin metabolites for up to 2 h postadministration.

### Metabolites of quercetin in human plasma

**Figure 4:**
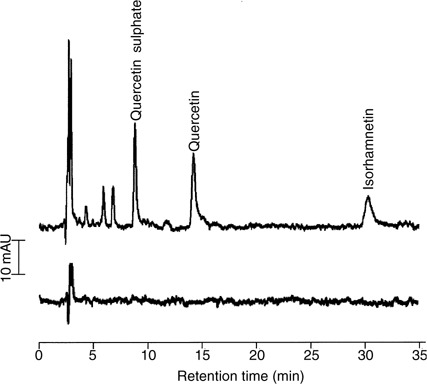
High-performance liquid chromatography chromatograms of extracts of plasma from a patient obtained before (bottom trace) and 5 min after administration (top trace) of quercetin (280 mg m^−2^ i.v.). Peaks were identified on the basis of cochromatography and selected ion monitoring MS, which afforded *m/z* 301 for quercetin, *m/z* 315 for isorhamnetin and *m/z* 381 for quercetin 3-*O*-sulphate. AU=absorbance units. For details of sample preparation and chromatographic and mass spectrometric analysis see Materials and Methods.

A patient with confirmed cancer received quercetin via the i.v. route. Extracts of plasma obtained just prior to, and 5 min after, drug administration were analysed by HPLC (Figure 4

) using an isocratic method, in contrast to the gradient elution procedure employed in the rat study described above. Analysis by MS of peak eluates confirmed the inferences for quercetin, isorhamnetin and quercetin 3′-*O*-sulphate ([M–H]^−^=*m/z* 381). When plasma from a rat, which had received quercetin, was analysed using the isocratic method, these three species were similarly prominent (data not shown).

### GSH conjugates of quercetin

**Figure 5:**
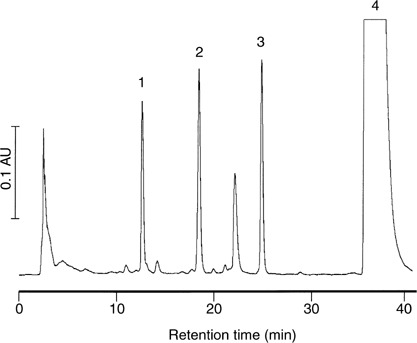
High-performance liquid chromatography chromatogram of an extract of a mixture of quercetin (1 mM), glutathione (1 mM) and mushroom tyrosinase (150 U ml^−1^). Mass spectral analysis performed in the selected ion monitoring mode suggests the following peak allocation: ‘1’ *bis*-glutathionyl-*S*-quercetin (*m/z* 911), ‘2’ and ‘3’ glutathionyl-*S*-quercetin (*m/z* 606) and ‘4’ quercetin. AU=absorbance units. For details of incubation conditions, sample preparation and chromatographic conditions see Materials and Methods. The chromatogram shown is representative of three separate experiments.

In the light of the nephrotoxic manifestations of quercetin observed in a clinical trial (Ferry *et al*, 1996), the hypothesis was tested such that quercetin might undergo metabolic oxidation in species reacting with GSH conjugation to furnish moieties that could potentially be targeted specifically to the kidney. To this end, quercetin was incubated with mushroom tyrosinase, which can oxidise catechols to their quinones (Duckworth and Coleman, 1970). High-performance liquid chromatography analysis of an extract of the reaction mixture furnished seven peaks eluting prior to quercetin (Figure 5

). Off-line HPLC–MS analysis afforded *m/z* 911 [M–H]^−^ for peak ‘1’, consistent with *bis*-glutathionyl-*S*-quercetin, while peaks ‘2’ and ‘3’ furnished *m/z* 606 [M–H]^−^, suggestive of glutathionyl-*S*-quercetin. Similar results were obtained when horseradish peroxidase was used as an oxidising agent instead of tyrosinase. Next, the hypothesis was tested such that GSH conjugates also occur in rats, which had received quercetin via the p.o. or i.v. routes. However, none of the species observed in the *in vitro* incubates (Figure 5) could be detected in extracts of plasma, urine, faeces or bile from rats. Additional experiments in which the catabolism of GSH conjugate species was inhibited by the coadministration of the *γ*-glutamyl transpeptidase inhibitor acivicin also failed to engender evidence for the formation of such conjugates (unpublished result). These results suggest that GSH conjugates of quercetin are not formed in the rat *in vivo* at levels detectable by the HPLC method employing UV and MS detection as described here. We cannot exclude the possibility that more sensitive detection methods such as electrochemical ones (Carvalho *et al*, 2004) may have led to quercetin GSH conjugate identification.

### Effect of quercetin metabolites on cellular PGE-2 production

**Figure 6:**
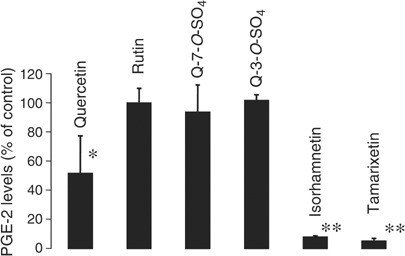
Effect of quercetin, rutin quercetin-7-*O*-sulphate (Q-7-*O*-SO_4_), quercetin-3-*O*-sulphate (Q-3-*O*-SO_4_), isorhamnetin and tamarixetin (each 10 *μ*M) on COX-2 enzyme activity, as reflected by PGE-2 levels, in cultures of HCA-7 colon cancer cells. The COX complement of HCA-7 cells is made up almost exclusively of COX-2, while COX-1 is present only to a minor extent (Sharma *et al*, 2001). Cells were incubated with agents for 6 h, and PGE-2 levels were measured by ELISA. Asterisks indicate that values were significantly different from controls (^*^P<0.01, ^**^P<0.001). For details of incubation conditions and measurement see Materials and Methods. The values are the mean±s.d. of three to five separate experiments, each conducted in triplicate.

Human-derived HCA-7 colon cancer cells contain significant levels of COX protein, which has been shown to be almost exclusively COX-2 (Sharma *et al*, 2001). Therefore, measurement of PGE-2 in the cellular supernatant reflects predominantly COX-2 levels and activity. Cells were incubated for 6 h with quercetin or with selected glycosides and metabolites, and PGE-2 levels were determined. The IC_50_ for inhibition of PGE-2 production by quercetin was found to be 10 *μ*M, and at 1 *μ*M quercetin reduced PGE-2 levels by 30% (result not shown). The quercetin progenitor glycoside rutin and the four quercetin metabolites isorhamnetin, tamarixetin, quercetin 3-*O*-sulphate and quercetin 7-*O*-sulphate were compared with quercetin in terms of their ability to inhibit PGE-2 production (Figure 6

). Cells were incubated with agents at 10 *μ*M. While rutin, quercetin 3-*O*-sulphate and quercetin 7-*O*-sulphate failed to affect PGE-2 levels, both isorhamnetin and tamarixetin decreased PGE-2 levels by more than 90%. It is important to point out that the COX-inhibitory activity of quercetin glucuronides, major quercetin metabolites in rats (Day *et al*, 2001), was not explored.

## DISCUSSION

The results described above allow the following four conclusions to be drawn as to the metabolism of quercetin when administered as authentic aglycone: (i) in the rat, quercetin undergoes metabolic methylation and multiple conjugation reactions with activated sulphate and glucuronide, and metabolically generated isorhamnetin is also subject to such secondary conjugation reactions; (ii) methylated and sulphated species are metabolites of quercetin in humans; (iii) while quercetin can be oxidised to species that undergo reaction with GSH under *in vitro* conditions, such species seem not to be generated at detectable levels in rats *in vivo*; (i.v.) metabolic methylation of quercetin confers potent COX-inhibitory properties onto the flavone molecule.

The pattern of conjugates generated in the rat as established here via scrupulous metabolite characterisation by MS is in broad agreement with previous reports in the literature on the biotransformation of quercetin after oral administration either as dietary glycoside or genuine aglycone (Ueno *et al*, 1983; Manach *et al*, 1997; Piskula and Terao, 1998). Only one methylated species, isorhamnetin (3′-*O*-methyl quercetin), was identified here, while its 4′-isomer, tamarixetin, was not. Tamarixetin was found previously in the plasma of rats, but only when they had received a flavonol-free diet prior to quercetin (Manach *et al*, 1997), which is consistent with its absence in the plasma of the rats studied here. Prior to our study, conjugates of quercetin had not been described in humans following the i.v. administration of pure quercetin. While in the work described here metabolism data were obtained from one human only, the results clearly hint at the possibility that quercetin undergoes metabolic sulphation and methylation not only in rodents but also in humans. Gross comparison of the doses and plasma levels suggests that the plasma levels achieved in rats immediately after i.v. injection resemble the levels achieved in the clinical trial of quercetin in humans (Ferry *et al*, 1996). The quercetin dose used in rats here in terms of body surface area (Freireich *et al*, 1966) was 40.6 mg m^−2^ (6.25 mg kg^−1^), approximately 4.5% of the dose given to humans (945 mg m^−2^). In analogy, the plasma level (14 *μ*M) achieved in rats 5 min after quercetin administration was approximately 5% of that found in humans immediately after quercetin administration, which was 280 *μ*M (Ferry *et al*, 1996).

In the pharmacokinetic phase I study in cancer patients, quercetin was well tolerated (Ferry *et al*, 1996), and there was evidence of pharmacodynamic activity as indicated by inhibition of epidermal growth factor receptor phosphorylation, yet indications of nephrotoxicity were also observed. Overall, the study led to the conclusion that poor bioavailability mitigates against the potential of quercetin as a clinically viable therapeutic agent. The multiple conjugation reactions that quercetin has been shown to undergo in the work described here and previously, leading to methylated, sulphated and glucuronidated flavones, may be responsible for, or contribute to, its poor systemic availability. Significantly, such conjugative metabolism does not seem to include reaction with GSH, thus rendering the possibility unlikely that GSH conjugates of quercetin are causally implicated in its putative nephrotoxic potential. In addition, there is no consistent conclusion to the effect of quercetin on cellular GSH levels by quercetin (Nijhoff *et al*, 1993, CanivencLavier *et al*, 1996; Sahu and Gray, 1996).

The two methylated congeners of quercetin, isorhamnetin and tamarixetin displayed potent COX enzyme inhibition, and their inhibitory potency was significantly higher than that of the parent molecule, quercetin. The IC_50_ for COX enzyme inhibition of quercetin has been reported to be 16 *μ*M (Formica and Regelson, 1995), and in the experimental design employed here using HCA-7 cells, it was approximately 10 *μ*M. The nature of the COX-inhibitory activity of the two metabolites and its relative specificity for COX-2 necessitates further investigation, as this observation may have clinical implications. That the methylated metabolites may conceivably play a pharmacodynamic role in the mammalian organism is borne out by a preliminary unpublished analysis of quercetin conjugates in bile conducted in our laboratory, according to which a major portion of drug-derived species generated in the rat organism was isorhamnetin.

The poor bioavailability of quercetin in the phase I clinical evaluation (Ferry *et al*, 1996) led to the conclusion that administration of quercetin may fail to furnish levels of bioactive species sufficient to exert useful pharmacological activity. This conclusion appears premature in the light of the fact that quercetin undergoes avid metabolism in species that may possess pharmacological activity, as hinted by our finding that isorhamnetin and tamarixetin are potent COX-2 inhibitors, at least in cells *in vitro*. Consistent with the notion that quercetin metabolites may be partially responsible for the pharmacological activity of the parent flavonol, quercetin conjugates have previously been found to retain, at least in part, the abilities of the parent molecule to exert antioxidation (Da Silva *et al*, 1998; Manach *et al*, 1998) and to inhibit xanthine oxidase and lipoxygenase (Day *et al*, 2000). Furthermore, products of the metabolic fission of quercetin have been shown to demonstrate greater antioxidant potency than their precursor (Merfort *et al*, 1996).

From the mechanistic standpoint, quercetin is a multitargeted agent and has poor bioavailability in the mammalian organism, and it shares these properties with many diet-derived polyphenols. The results outlined here support the supposition that metabolites of quercetin may be involved in its bioactivity, a suggestion that warrants further study designed to unravel the full potential of this molecule in the prevention or treatment of disease.
